# Characterization of Novel *Lachancea thermotolerans* Strains for Application in Table Olive Fermentation

**DOI:** 10.3390/foods15111883

**Published:** 2026-05-26

**Authors:** Patricia Gil-Flores, David Penco-Parra, Joaquín Bautista-Gallego

**Affiliations:** Departamento de Ciencias Biomédicas (Área de Microbiología), Facultad de Ciencias, Universidad de Extremadura, 06006 Badajoz, Spain

**Keywords:** non-*Saccharomyces*, *Lachancea*, table olives, oleuropein, fermentation, lactic acid, technological traits, stress tolerance

## Abstract

*Lachancea thermotolerans* is a non-*Saccharomyces* yeast characterized by its biotechnological potential, due, among other reasons, to its capacity to produce L-lactic acid and its impact on the organoleptic profiles of final fermented products. While its traits have been widely studied in fermented beverages and vinification, its potential use in other fermented foods, such as table olives, has been poorly studied. For this reason, the characterization and laboratory screening of *Lachancea thermotolerans* strains were performed in the present study. In this context, the potential use of forty *L. thermotolerans* strains in Spanish-style table olives was discussed based on pH and salt concentration tolerance, two key stress factors in the production process. Furthermore, the effect of oleuropein—the most abundant polyphenol in raw drupes—on L-lactic acid production was described to better understand and predict the behavior of *L. thermotolerans* under real conditions. In addition to stress resistance and L-lactic acid production, the suitability of the strains was assessed through their resistance to cycloheximide and copper, key indicators of tolerance to antimicrobial agents and agricultural residues. Finally, Principal Component Analysis of mixed data (PCAmix) and Hierarchical Clustering on Principal Components (HCPC) were performed to stratify and describe the intraspecific variability of this species, leading to the preselection of fifteen promising *L. thermotolerans* strains for use as starter cultures in Spanish-style table olive elaboration.

## 1. Introduction

Table olives represent one of the most important fermented vegetable products worldwide and constitute a cornerstone of the Mediterranean diet. According to the International Olive Council (IOC), global table olive production has experienced sustained growth during the last decades, exceeding 3 million tons annually, reflecting an increasing consumer demand for fermented and functional foods [1]. The economic and cultural relevance of table olives is particularly significant in Mediterranean countries, where olive cultivation and processing have been historically rooted. Spain is currently the leading producer and exporter of table olives worldwide, accounting for a substantial proportion of global production and commercialization [2]. Within Spain, regions such as Andalusia and Extremadura play a dominant role due to their favorable climatic conditions, where “Manzanilla” and “Gordal”, and “Manzanilla Cacereña”, respectively, are the most widely cultivated varieties in these regions. The socio-economic impact of table olive production in these regions is considerable, contributing significantly to rural employment and agro-industrial development [3].

The production of table olives relies mainly on spontaneous or controlled fermentation processes that ensure product preservation, safety, and sensory quality. During fermentation, complex microbial consortia develop and drive the biochemical transformations responsible for debittering, acidification, and flavor formation. Traditionally, lactic acid bacteria (LAB) have been considered the main microorganisms responsible for successful olive fermentation due to their ability to produce organic acids and lower pH, thus preventing the growth of spoilage and pathogenic microorganisms [4,5]. However, the fermentation process is often influenced by numerous factors, including salt concentration, temperature, oxygen availability, and the initial microbial population present on the fruit surface and processing environment. In spontaneous fermentations, the absence of controlled inoculation leads to highly variable microbial dynamics, which may result in fermentation failures, gas pocket formation, off-flavors, or textural defects, ultimately affecting product quality and economic profitability. In this context, starter cultures have been increasingly applied in food fermentations as a means to improve process control, enhance reproducibility, and ensure predictable product characteristics. Their use allows the dominance of selected microorganisms with known technological properties, reducing variability associated with spontaneous microbial successions [6,7]. In addition, current research has focused on the selection of starter cultures with specific functional traits, such as stress tolerance or targeted metabolic activities, to further improve fermentation performance and product quality [5,6].

While LAB have garnered the most attention, the role of yeasts in table olive fermentation is increasingly recognized as pivotal. Yeasts are naturally present on olive surfaces and are highly adapted to the stressful conditions of brine fermentation, such as high salinity and a broad pH range, from alkaline conditions at the beginning to acidic conditions at the end of fermentation. These microorganisms contribute positively to fermentation and facilitate nutrient availability and interactions with LAB, stimulating bacterial growth [8,9]. Certain yeast species, including *Lachancea thermotolerans*, have attracted increasing scientific interest due to their technological and functional properties [10]. This species has been reported to produce lactic acid, contribute to pH reduction, and generate desirable volatile organic compounds, which may improve fermentation stability and organoleptic quality in winemaking [11,12]. The combination of both *L. thermotolerans* and LAB activity may potentially lead to a faster acidification of the brine, and consequently to reaching the food safety pH threshold (<4.2). Furthermore, specific indigenous strains exhibit functional traits such as probiotic potential, antioxidant activity, and enzymatic capabilities (e.g., lipase and phytase) that improve nutrient bioavailability and texture preservation [13]. Nevertheless, the technological performance of yeast populations is highly strain-dependent, highlighting the need for the isolation and characterization of indigenous strains adapted to specific fermentation environments.

Overall, the primary objective of the present study was to perform a laboratory screening of a collection of *L. thermotolerans* strains for traits potentially relevant to table olive fermentation. This research evaluates key attributes, including metabolic efficiency in lactic acid production, both in the presence and absence of oleuropein, the main polyphenol present at the beginning of table olive fermentations, as well as tolerance to typical table olive fermentation stressors at initial stages, such as high salinity and high pH. Furthermore, this study assesses strain-specific resistance to chemical inhibitors, including copper and cycloheximide, which are critical markers for selecting robust strains for their potential use at industrial level. The comprehensive characterization of these strains provides essential insights into their suitability as starter cultures.

## 2. Materials and Methods

### 2.1. Yeast Strains

The biological material for this study comprised forty *Lachancea thermotolerans* strains belonging to the Table Olive Microorganism Collection at the University of Extremadura. These strains were isolated and taxonomically identified between 2024 and 2025 from a variety of natural ecosystems and agro-industrial niches (Table S1). In addition, a *Wickerhamomyces anomalus* strain isolated from Spanish-style table olive fermentation was incorporated as a representative microorganism isolated from industrial table olive fermentations. All strains were analyzed in duplicate for each assay to ensure reproducibility and data integrity.

All strains were stored at −80 °C in Yeast Extract Peptone Dextrose (YEPD, 2% glucose, 2% peptone and 1% yeast extract, *w*/*v*) broth medium with 20% glycerol (*v*/*v*) until their use. Thus, each strain was inoculated into 1 mL of YEPD broth medium and incubated at 30 °C. A 48 h incubation period was strictly maintained, allowing the microbial populations to reach their initial stationary growth phase prior to their deployment in the subsequent screening protocols, in order to standardize the physiological state of the inocula and enhance tolerance to subsequent stress conditions.

### 2.2. L-Lactic Acid Production

The concentration of L-lactic acid present in the biological samples was determined as described by Ferrando et al. [14]. Briefly, and to prevent D-glucose from becoming a limiting factor in the glycolysis reaction, YEPD broth medium was supplemented with D-glucose to a final concentration of 23% (*w*/*v*). In addition, the potential effect of the presence of oleuropein in L-lactic acid production was also studied. For this reason, a second modified rich YEPD broth medium was supplemented with 0.2% oleuropein (*w*/*v*).

Each strain was inoculated at 2% (*v*/*v*) in 14 mL of each aforementioned medium and kept at 30 °C for 48 h. All quantifications were performed in duplicates. Samples were analyzed by using a specific enzymatic kit (TDI) with a Miura One Multianalyzer (TDI, Barcelona, Spain). In this assay, the enzyme L-lactate dehydrogenase (L-LDH) catalyzes the oxidation of L-lactic acid to pyruvate with the concomitant reduction of nicotinamide adenine dinucleotide (NAD+). The increase in absorbance at 340 nm due to NADH formation is directly proportional to the concentration of L-lactic acid in the sample.

With the purpose of describing and visualizing the loss of L-lactic acid production by yeast strains due to the presence of oleuropein in the growth media, a raincloud plot was generated by using raincloud_1×1_repmes() from the raincloudplots package (v0.2.0) [15].

### 2.3. Comparative Tolerance to Sodium Chloride and pH Levels

#### 2.3.1. NaCl and pH Effects on Microbial Growth

Two NaCl concentrations (0 and 10%, *w*/*v*) and two pH levels (6 and 9) were tested using YEPD broth (2% glucose, 2% peptone and 1% yeast extract, *w*/*v*) as the base medium. Each variable was studied independently. Briefly, each well of a round-bottom 96-well plate was filled with 197 µL of culture medium and then inoculated with 3 µL of pre-cultured strain. The inoculum level was previously evaluated in our experimental system to ensure that cell growth remained within the detection range of the spectrophotometer, allowing accurate monitoring of growth dynamics. Growth curves for each yeast strain and treatment were constructed using optical density (OD) measures taken using a TECAN infinite 200Pro^®^ multiplate reader (Tecan, Männedorf, Switzerland) at 600 nm and room temperature. Measurements were taken every two hours over a period of five days, with shaking prior to each reading. The inocula consistently remained above the detection limit of the automatized spectrophotometer, as determined by comparison with a previously established calibration curve. Growth curve data were normalized by subtracting the value from the non-inoculated culture medium (control curve). All experiments were performed in duplicate.

#### 2.3.2. NaCl and pH Tolerance Comparison

Salt (*w*/*v*) and pH tolerance among yeast strains were assessed by calculating areas under the curves (*AUC*s). This parameter can be interpreted as biological fitness of a yeast strain in a specific condition, and can be calculated as

(1) $$\mathrm{AUC}=\sum_{i=1}^{n-1}\left(\frac{y_{i}+y_{i+1}}{2}\right)\left(t_{i+1}-t_{i}\right)$$

where *AUC* represents the area under the curve; *i* represents the index for a specific time interval; *n* is the total number of observations; *y_i_* and *y*_*i*+1_ are the optical density (OD) interpolated at two consecutive time points; and *t*_*i*+1_ and *t_i_* represent time values of the interval.

To compare salinity resistance, *AUC* was calculated for the control (without NaCl) and the 10% (*w*/*v*) salt, applying the trapezoid method via linear interpolation. Then, the relative diminution percent of OD was calculated as an estimation of biological viability and salt tolerance of each strain of the study.

(2) $${\mathrm{AUC}}_{0}=\sum_{i=1}^{n-1}\left(\frac{y_{i,0}+y_{i+1,0}}{2}\right)\left(t_{i+1}-t_{i}\right)$$

(3) $${\mathrm{AUC}}_{10}=\sum_{i=1}^{n-1}\left(\frac{y_{i,10}+y_{i+1,10}}{2}\right)\left(t_{i+1}-t_{i}\right)$$

(4) $$\Delta {\mathrm{AUC}}_{\mathrm{salt}}\left(\%\right)=\left(\frac{{\mathrm{AUC}}_{0}-{\mathrm{AUC}}_{10}}{{\mathrm{AUC}}_{0}}\right)\times 100$$

where *AUC*_0_ and *AUC*_10_ represent areas under the curve for 0% and 10% (*w*/*v*) NaCl, respectively; *y_i_*_,0_ and *y_i_*_,10_ represent OD values at time *t_i_* for 0% and 10% (*w*/*v*) NaCl, respectively; *t*_*i*+1_ and *t_i_* represent the time interval between consecutive observations; and Δ*AUC*_salt_ (%) represents the percentage decrease in AUC between 0% and 10% (*w*/*v*) NaCl.

To compare pH resistance, *AUC* was calculated for pH levels of 6 and 9, applying the trapezoid method via linear interpolation. Then, the relative diminution percent of OD was calculated as an estimation of biological viability and pH tolerance of each strain of the study.

(5) $${\mathrm{AUC}}_{6}=\sum_{i=1}^{n-1}\left(\frac{y_{i,6}+y_{i+1,6}}{2}\right)\left(t_{i+1}-t_{i}\right)$$

(6) $${\mathrm{AUC}}_{9}=\sum_{i=1}^{n-1}\left(\frac{y_{i,9}+y_{i+1,9}}{2}\right)\left(t_{i+1}-t_{i}\right)$$

(7) $$\Delta {\mathrm{AUC}}_{\mathrm{pH}}\left(\%\right)=\left(\frac{{\mathrm{AUC}}_{6}-{\mathrm{AUC}}_{9}}{{\mathrm{AUC}}_{6}}\right)\times 100$$

where *AUC*_6_ and *AUC*_9_ represent areas under the curve at pH 6 and 9, respectively; *y*_*i*,6_ and *y*_*i*,9_ represent OD values at time *t_i_* at pH 6 and 9, respectively; *t*_*i*+1_ and *t_i_* represent the time interval between consecutive observations; and Δ*AUC*_pH_ (%) represents the percentage decrease in *AUC* between pH 6 and 9.

The linear interpolation was applied by using map() and approxfun() functions from the Purr package (v1.2.1) [16]). Bar plots of relative diminution percent were generated by using ggplot2 package (v4.0.2) [17].

### 2.4. Qualitative Screening of Yeast Technological Traits

Differences in tolerance among *L. thermotolerans* strains were evaluated by inoculating 20 µL of each one in YEPD agar plates supplemented with 2 µg/mL cycloheximide or SD-agar plates (2% glucose, 0.67% yeast nitrogen base) supplemented with 60 mg/L CuSO_4_. Pre-cultures obtained as described in Section 2.1 were washed, serially diluted (10-fold) in 0.9% (*w*/*v*) saline solution, and spotted onto agar plates (5 µL per spot). Plates were read after 3–5 days of incubation at 28 °C in the dark.

### 2.5. Statistical Analysis

To better understand the intraspecific variability of *L. thermotolerans* and elucidate the possibility of stratification and selection of the most promising strains, Principal Component Analysis for mixed data (PCAmix) and Hierarchical Clustering were performed. In this analysis, the main quantitative variables were the percentage decrease in AUC between pH 6 and 9, the percentage decrease in AUC between 0% and 10% (*w*/*v*) NaCl, and the production of L-lactic acid and production of L-lactic acid in the presence of oleuropein, while cycloheximide and copper resistances were handled as discrete binary variables. Hierarchical Clustering of Principal Components (HCPC) was applied to the coordinates from the first two components of the PCAmix with the aim of identifying and grouping *L. thermotolerans* strains based on phenotypic similarities. Ward’s minimum variance method and Euclidean distance were performed with the purpose of ensuring an optimum cluster separation.

The PCAmixdata package (v3.1) [18] was used for multivariate analysis performance, and the FactoMinerR package (v2.13) [19] was used for clustering interpretation. A biplot with confidence ellipses (level = 0.68) and variable contribution segment visualization was generated using the factoextra package (1.0.7) [20] and ggplot2 package (v4.0.2) [17].

In addition, all data analysis in the present study was performed using R version 4.4.2 and RStudio version 2024.12.1. For statistical contrast between the variables of the clusters previously obtained, Shapiro–Wilk and Levene’s tests were first applied for assessing normality and homogeneity of variance assumptions, respectively. One-way ANOVA and Tukey’s HSD post hoc tests were applied when parametric assumptions were met, while Kruskal–Wallis and Dunn’s tests were applied when at least one of the clusters’ variables violated these assumptions. The statistical workflow was implemented utilizing the aov(), kruskal.test() and TukeyHSD() functions from the stats package (v4.4.2) for parametric comparisons, while the car (v3.1.5) [21] and FSA (v0.10.1) [22] packages were employed for leveneTest() and dunnTest(), respectively. On the other hand, statistical contrasts were visualized via violin plots with significance stars generated with the ggplot2 (v4.0.2) [16] and the ggpubr (v0.6.2) [23] packages.

## 3. Results

### 3.1. L-Lactic Acid Production and Oleuropein Influence

**Figure 1:**
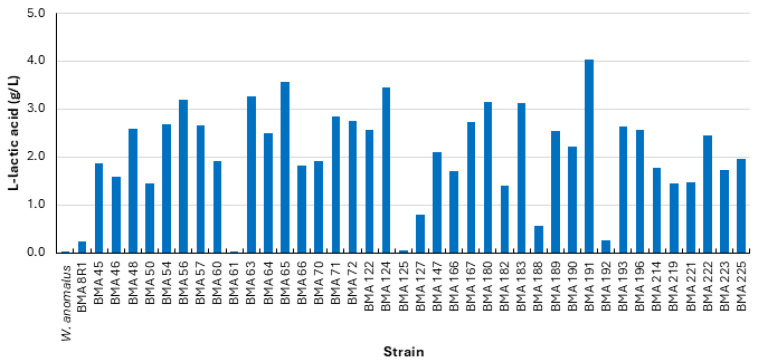
L-lactic acid production of each *L. thermotolerans* strain in YEPD broth medium supplemented with 23% glucose (*w*/*v*) after 48 h. Data are the mean values of two replicates. Standard deviation was consistently below 5% of the means.

Regarding L-lactic acid production, most strains produced higher levels than the reference *Wickerhamomyces anomalus*, typically associated with Spanish-style and brine-fermented green table olive fermentations (Figure 1). Notably, BMA 191 reached the highest level (4.03 g/L) after only 48 h, representing a more than 100-fold increase over *W. anomalus* (0.03 g/L). Other high-performing candidates included BMA 56 (3.19 g/L), BMA 63 (3.27 g/L), BMA 65 (3.58 g/L), BMA 124 (3.45 g/L), BMA 180 (3.16 g/L), and BMA 183 (3.13 g/L). On the other hand, some strains displayed low L-lactic acid production, such as BMA 8R1, BMA 61, BMA 125 and BMA 192. However, it must be emphasized that 85% of the studied strains were able to produce more than 1 g/L of L-lactic acid after only 48 h.

**Figure 2:**
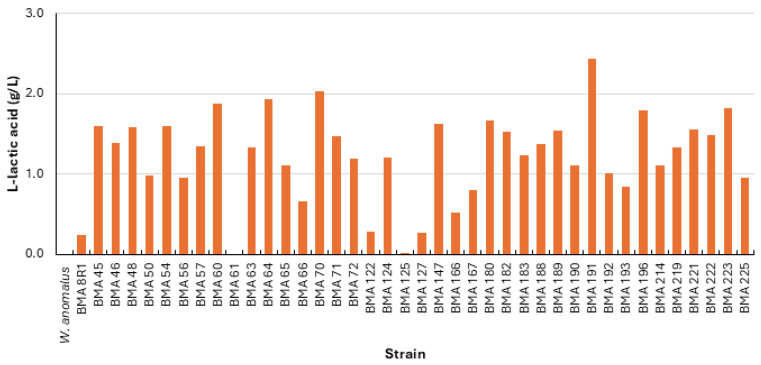
L-lactic acid production of each *L. thermotolerans* strain in YEPD broth medium supplemented with 23% glucose (*w*/*v*) and 0.2% oleuropein (*w*/*v*) after 48 h. Data are the mean values of two replicates. Standard deviation was consistently below 7% of the means.

The inclusion of oleuropein in the medium provided further differentiation. *W. anomalus* showed no detectable L-lactic acid production in the presence of oleuropein, thereby confirming its metabolic incapacity to synthesize this organic acid under both conditions (Figure 2). In contrast, some strains such as BMA 191 maintained robust metabolic activity, producing the highest levels (2.44 g/L), although lower than without the polyphenol presence. On the other hand, some strains showed the same behavior with very low levels, such as BMA 8R1, BMA 61, BMA 125 and BMA 127.

**Figure 3:**
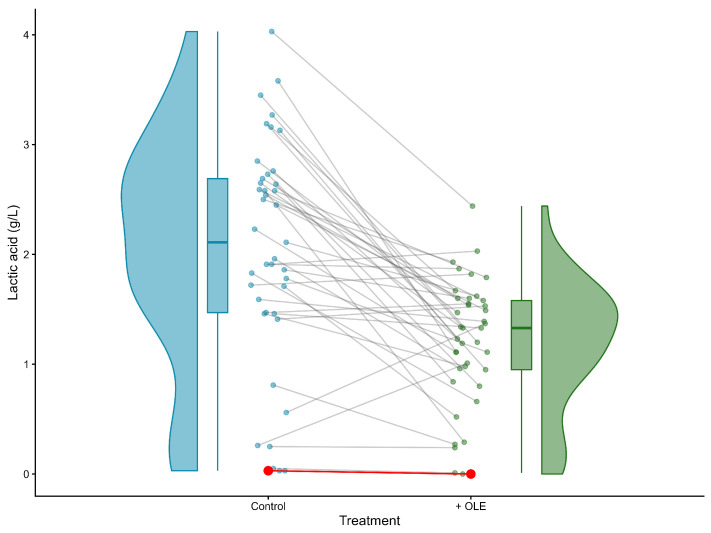
Raincloud plot comparing behaviors of *Lachancea thermotolerans* strains with and without the addition of oleuropein. Reference *W. anomalus* strain in red.

Comparison of both performances (Figure 3) reveals three distinct behaviors depending on the trend in lactic acid production. Firstly, the predominant group, composed of more than half of the strains tested, displayed a reduction in L-lactic acid production due to the presence of oleuropein. Secondly, thirteen strains had a very stable production in both conditions, showing similar levels with and without this stressful compound. Lately, two strains (BMA 188 and BMA 192) showed a sharp increase in production in the presence of oleuropein. These last two groups exhibited high resilience, which may indicate higher tolerance to oleuropein under the tested laboratory conditions.

### 3.2. Technological Traits

For further biotechnological characterization, all *L. thermotolerans* strains were evaluated for their resistance to copper and cycloheximide. Strain-dependent behavior was observed for both qualitative analyses (Table 1). Even more, the same analyses were performed after 100 doublings for each strain in order to evaluate possible genetically unstable resistant strains for each compound. No changes were detected after 100 doublings for all strains.

### 3.3. Halotolerance Response

**Figure 4:**
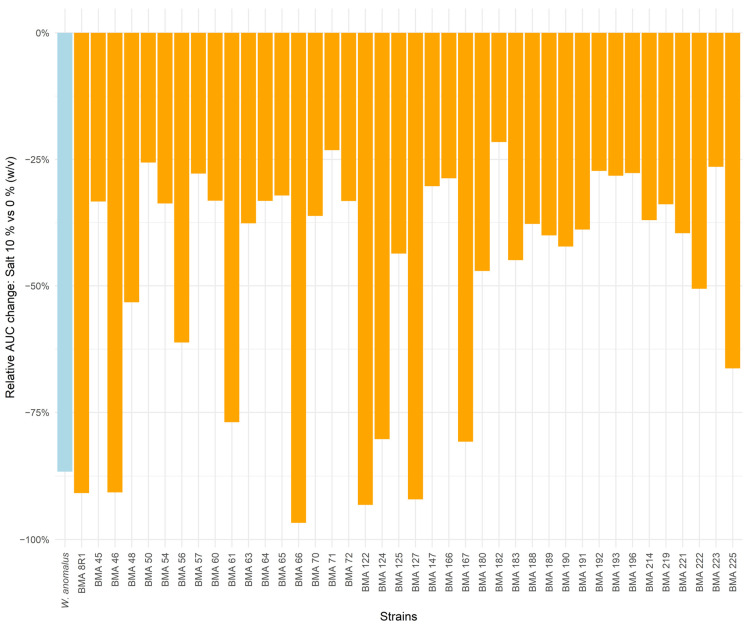
Tolerance performance of *Lachancea thermotolerans* strains, measured through a comparative approach using the AUC between 0 g/L and 100 g/L of sodium chloride (*w*/*v*). Data are the mean values of two replicates. Standard deviation was consistently below 6% of the means.

The salt resistance assay, measured as the relative change in the *AUC* between 0 and 10% (*w*/*v*) NaCl, further highlighted the superior robustness of the strains. The reference strain, *W. anomalus*, suffered a severe growth inhibition of 86.53% (Figure 4). Crucially, a substantial group of strains showed a markedly better response to high salinity typically found in the first stages of table olive fermentations. Several strains displayed the best performance, with an *AUC* reduction lower than 30%, such as BMA 50, BMA 57, BMA 71, BMA127, BMA 147, BMA 182, BMA 192, BMA 193, BMA 196 and BMA 223. This behavior demonstrated the highest halotolerance of these strains, maintaining a significantly higher general growth than the *W. anomalus* and suggesting that these strains may be better for industrial brine conditions, where osmotic stress is a limiting factor for traditional starters.

### 3.4. Effect of pH on Microbial Growth

**Figure 5:**
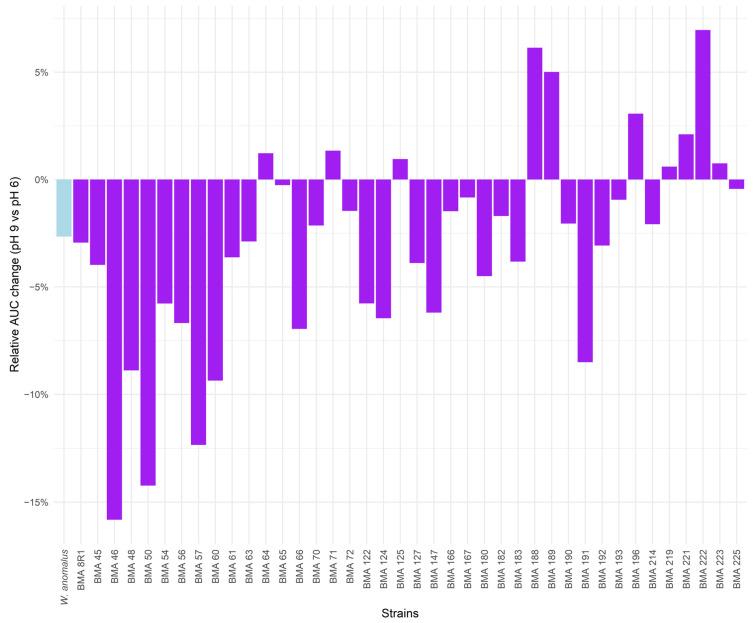
Tolerance performance of *Lachancea thermotolerans* strains, measured through a comparative approach using the *AUC* between pH 6 and pH 9. Data are the mean values of two replicates. Standard deviation was consistently below 7% of the means.

The adaptation of the studied strains to the alkaline conditions typical of the early stages of Spanish-style table olive fermentation (pH 9.0) was assessed by comparing the *AUC* relative to the actual industry inoculation level (close to pH 6.0) [24]. The response to alkaline stress was highly strain-dependent (Figure 5), with most isolates exhibiting slight changes in their growth capacity.

While a significant amount of the strain collection showed a decrease in *AUC* under alkaline conditions, several strains demonstrated a remarkably better ability to thrive at pH 9.0. Specifically, BMA 188, BMA 189, BMA 196 and BMA 222 exhibited a significant positive relative *AUC* change, suggesting that these strains are not only tolerant but may even be favored by the initial alkaline environment of the brine. In contrast, the reference strain *W. anomalus* showed a slight sensitivity to alkaline stress, with a reduction in growth performance. This pattern was also observed for many of the *L. thermotolerans* strains. On the other hand, BMA 46, BMA 50 and BMA 57 showed the worst performance due to the effect of high pH levels, obtaining a reduction in growth higher than 10%.

These findings, together with the previously described salt and phenolic tolerance, further categorize strains such as BMA 188 and BMA 222 as yeasts that may be promising candidates for further validation in model olive brines.

### 3.5. Principal Component Analysis of Mixed Data

**Figure 6:**
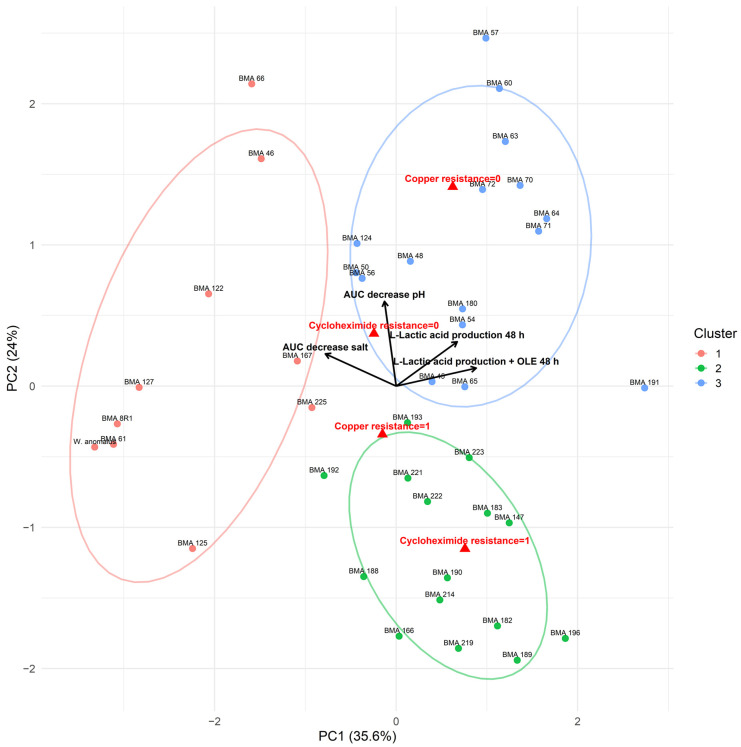
Principal Component Analysis of mixed data based on standardized data from quantitative and qualitative parameters studied for all *Lachancea thermotolerans* strains, including the projection of the variables and strains onto the plane formed by the two first dimensions.

Both the quantitative and qualitative data obtained for all *L. thermotolerans* strains were subjected to PCAmix (Figure 6). This approach was applied to assess the presence of groups with key characteristics related to technological traits and survival to stress conditions. The first two principal dimensions explained 35.6% and 24.0% of the total variability, respectively.

The first dimension (35.6%) was strongly influenced by both conditions for L-lactic acid production and resistance to NaCl (Figure S1). These results suggest that the ability to produce this organic acid and the resistance to 10% NaCl (*w*/*v*) represent the main source of variation among groups. In contrast, the second dimension (24%) was dominated by qualitative technological characteristics. In this dimension, resistance to cycloheximide and sensitivity to copper showed the highest contributions. However, the contribution of the variable pH had the lowest impact over both principal components. This fact indicated that yeast strain behavior remains relatively uniform or slightly changes under high pH levels, highlighting the great performance of almost all strains in typically stressful conditions at the first stages of table olive fermentations.

Hierarchical Clustering was applied to the PCAmix results, enabling the identification of three different clusters (Figure 6). Cluster 1 comprised nine *L. thermotolerans* strains characterized by a low tolerance to NaCl. Furthermore, *W. anomalus* was placed in cluster 1, highlighting the better performance of the *L. thermotolerans* strains of the other two clusters. In contrast, cluster 2 included fifteen yeast strains that presented the best global characteristics for all variables, showing a high degree of phenotypic plasticity and metabolic robustness. Lastly, cluster 3 comprised sixteen strains with a good production of L-lactic acid but a reduction in growth in alkaline conditions. This phenomenon may constitute a rate-limiting factor in competitive fermentations due to the pH conditions of Spanish-style table olive processing.

**Figure 7:**
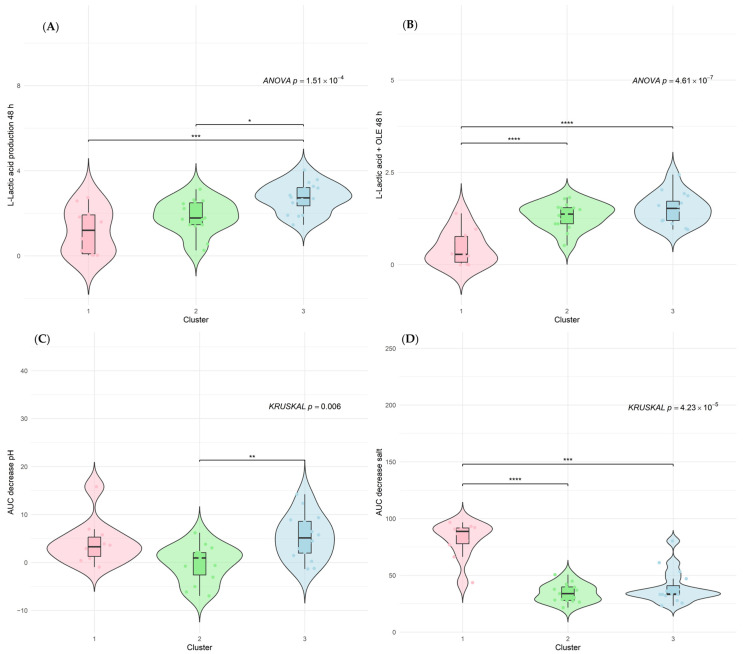
Violin plots for statistical differences between variables of clusters generated in HCPC analysis. (**A**) L-lactic acid production in the absence of oleuropein; (**B**) L-lactic acid production in the presence of oleuropein; (**C**) percentage change in *AUC* under pH conditions; (**D**) percentage change in *AUC* under NaCl conditions. Statistical significance between experimental groups was obtained using Dunn’s post hoc test and Bonferroni correction. Significance levels are indicated as follows: *p* ≤ 0.05 (*), *p* ≤ 0.01 (**), *p* ≤ 0.001 (***), and *p* ≤ 0.0001 (****).

To further corroborate the clusters formed in the last analysis, statistical tests between the variables of the clusters were performed, taking into account normality assumptions (Figure 7). With respect to L-lactic acid production without oleuropein, slightly significant differences appeared between clusters 1 and 2, followed at a distance by cluster 3, which showed a significantly lower production than the others (Figure 7A). Interestingly, cluster 2, the most promising one, exhibited a lower performance for L-lactic production, but, when oleuropein was considered, there were no statistical differences between this one and cluster 3, matching its capacity to generate this acid (Figure 7B). In contrast, cluster 1 was still the group with the least L-lactic production. With respect to pH and salt *AUC* percent diminution, cluster 2 displayed a lower reduction in biological fitness under the two stressors, followed only by cluster 3, which did not show a significant decrease in the area under the curve regarding NaCl concentration (Figure 7C,D). Considering these tests, cluster 2 remained the most promising group due to its phenotypic plasticity under the key stress conditions tested in this study.

## 4. Discussion

The growing interest in *L. thermotolerans* as a functional yeast in food fermentations is largely driven by its unique capacity to produce lactic acid during sugar metabolism, a metabolic feature that distinguishes it from most conventional yeasts and functionally aligns it with lactic acid bacteria (LAB). This heterolactic behavior has been extensively described in recent years, particularly in wine biotechnology, where *L. thermotolerans* contributes to biological acidification, microbial stability, and sensory modulation [25,26,27]. More recent studies have further confirmed its role as a “bio-acidifier” yeast, highlighting its capacity to modulate pH and total acidity in a predictable and strain-dependent manner [28,29].

In the present study, *W. anomalus* was used as a control because it is one of the most representative yeast species in Spanish-style table olive fermentations. However, although other yeast species are also commonly found in this process, none of them are known to produce L-lactic acid. Some *L. thermotolerans* strains significantly outperformed the reference *W. anomalus*. Notably, strain BMA 191 showed an exceptional increase in L-lactic acid production, reaching 4.03 g/L after 48 h. Recent studies have highlighted the potential of non-Saccharomyces yeasts to modulate acidity and influence fermentation dynamics, supporting their use as biotechnological tools in controlled fermentation processes [30].

The influence of oleuropein represents a key aspect of this study, as phenolic compounds are among the main selective pressures in olive brines. Oleuropein and its derivatives, such as hydroxytyrosol, have well-documented antimicrobial properties, particularly against LAB and certain yeasts [31]. In agreement with previous reports, the presence of oleuropein reduced metabolic activity in a substantial proportion of strains. However, the ability of several *L. thermotolerans* isolates to maintain or even enhance lactic acid production under these conditions suggests the presence of adaptive responses to phenolic stress. The ability of the best lactic acid producers to perform under phenolic compound stress reinforces the potential of *L. thermotolerans* as a candidate for starter culture development beyond the wine sector, extending its application to vegetable fermentation, where early pH reduction is a key factor for inhibiting spoilage microbiota and ensuring product safety [5].

Recent research has highlighted that yeasts associated with olive fermentations often possess enzymatic systems, such as β-glucosidases and esterases, involved in the transformation of phenolic compounds [32,33]. More recent works have also demonstrated that non-*Saccharomyces* yeasts can actively modulate phenolic profiles, contributing not only to detoxification but also to flavor development [34]. In line with these observations, the observed increase in lactic acid production in the presence of oleuropein for certain strains (e.g., BMA 188 and BMA 192) may reflect inducible metabolic pathways or co-metabolism strategies, where phenolic degradation products are partially assimilated or trigger stress responses that redirect metabolic fluxes. Such phenotypes are particularly valuable for directly brined olives or early fermentation stages in Spanish-style processing, where phenolic concentrations are high.

Salt tolerance is another critical determinant for the successful application of starter cultures in olive fermentations. The ability of several *L. thermotolerans* strains to maintain growth under high NaCl concentrations (up to 10% *w*/*v*) clearly distinguishes them from the reference strain and highlights their technological potential. Osmotic stress is known to induce complex cellular responses in yeasts, including glycerol accumulation, activation of the high-osmolarity glycerol (HOG) pathway, and membrane remodeling [35,36]. In the context of olive fermentations, halotolerance has been identified as a key trait for microbial dominance and persistence [5,9]. Recent studies have further emphasized that yeast strains isolated from high-salt environments often exhibit cross-protection mechanisms that also enhance tolerance to other stresses, such as ethanol or oxidative stress [35], which may explain the overall robustness observed in some of the strains evaluated here.

The ability to grow under alkaline conditions represents an additional competitive advantage, particularly in Spanish-style table olive processing. Following lye treatment, the pH of the brine can reach values close to 9.0, creating a hostile environment for many microorganisms. In this study, several strains (e.g., BMA 188, BMA 196, and BMA 222) not only tolerated but even showed improved growth under alkaline conditions, which makes these strains potential candidates for carrying out table olive fermentation from earlier stages, although further investigation should ensure the survival of *L. thermotolerans* in real fermentation conditions. This phenotype has been rarely described in fermentative yeasts but is increasingly recognized as an adaptive trait in microorganisms associated with olive ecosystems [9]. More recent studies on microbial ecology in table olives have confirmed that early-stage fermentations select for highly stress-resistant species capable of coping simultaneously with high pH, salinity, and phenolic content [4,37]. Therefore, the use of “alkaline-resilient” *L. thermotolerans* strains could facilitate rapid colonization of the brine environment, reducing the lag phase and improving process control.

Another important aspect highlighted in this study is the phenotypic stability of the evaluated strains. The absence of changes after 100 doublings in the presence of copper and cycloheximide indicates that these resistance traits are stable and not easily lost during propagation. This is a crucial requirement for industrial starter cultures, as stability ensures reproducibility and reliability across fermentation batches. Copper tolerance is highly relevant due to the widespread use of copper-based fungicides in Mediterranean agriculture, which can lead to its accumulation in fermentation environments [38]. Accordingly, tolerance to inhibitory compounds is considered a key technological trait in yeast selection for table olive fermentation [39]. Recent studies have also emphasized the importance of selecting starter cultures with stable stress-resistance traits to ensure consistent performance under industrial conditions [40].

The multivariate analysis performed in this work provides an integrative framework for understanding the phenotypic diversity of *L. thermotolerans*. The identification of lactic acid production and salt tolerance as the main drivers of variability is consistent with their central role in fermentation performance [41]. The clustering analysis further revealed the existence of a group of strains (cluster 2) combining high metabolic activity with strong resistance to multiple stress factors. This type of “multifunctional” phenotype is increasingly recognized as essential for the development of effective starter cultures in complex fermentation systems [34]. In contrast, the identification of strains with high acidification capacity but sensitivity to alkaline pH (cluster 3) highlights the importance of multi-trait selection strategies. Recent approaches in food microbiology emphasize the need to evaluate starter cultures under conditions that closely mimic real industrial environments, considering the simultaneous action of multiple stressors [37].

Overall, the results of this study suggest that the tested *L. thermotolerans* exhibits remarkable phenotypic plasticity and adaptability to the harsh conditions characteristic of table olive fermentations. The combination of L-lactic acid production, tolerance to phenolic compounds, halotolerance, and alkaline adaptability highlights this species as a highly promising candidate for the development of next-generation starter cultures. This aligns with current trends in food biotechnology, where non-*Saccharomyces* yeasts are increasingly exploited to improve process control, safety, and product quality [27,29,42].

Future work should focus on validating these findings at pilot and industrial scales, as well as on elucidating the interactions between *L. thermotolerans* and LAB during co-fermentation via metagenomic approaches. Recent studies highlight the importance of yeast–bacteria consortia in shaping fermentation dynamics and product characteristics [34]. In some cases, synergistic interactions between yeasts and lactic acid bacteria have been reported to improve fermentation performance and product quality, representing a promising avenue for further research in table olive biotechnology. There are also studies that reveal the polygenic nature of the lactate dehydrogenase enzyme, which can be activated via distinct transcription factors under differential media-dependent conditions. Further studies related to the impact of table olive stressor factors on the expression of these genes would be necessary to uncover the nature of the lactate dehydrogenase pathway in the production of this fermented food and the possibility of potentiating L-lactic acid production [43].

## 5. Conclusions

The present study demonstrates the significant potential of *L. thermotolerans* as a starter culture for table olive fermentation. Its remarkable tolerance to high salinity and alkaline pH conditions facilitates early-stage inoculation, potentially enabling its dominance throughout the fermentative process. Notably, the most distinctive attribute identified in specific strains is the production of L-lactic acid in the presence of oleuropein. This metabolic capability may contribute to pH reduction, but its effect under real olive fermentation conditions requires further validation.

The observed potential of *L. thermotolerans* to influence fermentation-related parameters suggests that selected strains may have potential technological relevance in reducing spoilage or alteration risks. These findings indicate that selected strains could be considered promising candidates for table olive fermentation, although their actual impact on fermentation kinetics and processing time remains to be confirmed under pilot-scale fermentation conditions. Although current industry trends primarily favor LAB or multi-species consortia, these findings suggest that *L. thermotolerans* can be effectively deployed both as a stand-alone starter culture and in combination with LAB.

Future research should focus on validating the performance of selected *L. thermotolerans* strains in industrial-scale table olive fermentations. Further investigation is required to characterize its interaction with LAB and its ability to form biofilms on the olive surface, a well-documented trait in species such as *Candida boidinii*. For this reason, the selected strains should be tested in a model olive brine in the presence of LAB, with measurements of pH, organic acids, microbial counts, and sensory characteristics. Additionally, the integration of metagenomic approaches will be essential to accurately assess microbial dynamics and yeast dominance at each stage of the process.

## Figures and Tables

**Table 1:** Phenotypic characterization of *Lachancea thermotolerans* strains. Cycloheximide resistance was tested on YEPD_CYH_ supplemented with 2 μg/mL cycloheximide. Resistance to copper was tested on SD-agar plates supplemented with 60 mg/L CuSO_4_.

Strain	YEPD_CYH_	SD_Cu_
*W. anomalus*	S	R
BMA 8R1	S	R
BMA 45	S	R
BMA 46	S	R
BMA 48	S	R
BMA 50	S	R
BMA 54	S	R
BMA 56	S	R
BMA 57	S	S
BMA 60	S	S
BMA 61	S	R
BMA 63	S	S
BMA 64	S	S
BMA 65	S	R
BMA 66	S	S
BMA 70	S	S
BMA 71	S	S
BMA 72	S	S
BMA 122	S	R
BMA 124	S	R
BMA 125	S	R
BMA 127	S	R
BMA 147	R	R
BMA 166	R	R
BMA 167	S	R
BMA 180	S	R
BMA 182	R	R
BMA 183	R	R
BMA 188	S	R
BMA 189	R	R
BMA 190	R	R
BMA 191	R	R
BMA 192	S	R
BMA 193	S	R
BMA 196	R	R
BMA 214	R	R
BMA 219	R	R
BMA 221	S	R
BMA 222	S	R
BMA 223	S	R
BMA 225	S	R

S: sensitive, total growth inhibition after 48 h; R: resistant, detected growth after 48 h.

## Data Availability

The original contributions presented in the study are included in the article/Supplementary Material, further inquiries can be directed to the corresponding author.

## Supplementary Materials

The following supporting information can be downloaded at: https://www.mdpi.com/article/10.3390/foods15111883/s1, Figure S1: Variable contributions to the first two dimensions of PCA for *Lachancea thermotolerans* characterization. Table S1: Microorganisms used in this study and their ecological origin and geographic location.

## Abbreviations

The following abbreviations are used in this manuscript:

LAB	Lactic Acid Bacteria
YEPD	Yeast Extract Peptone Dextrose
AUC	Area Under the Curve
OD	Optical Density
PCAmix	Principal Component Analysis of Mixed Data
HCPC	Hierarchical Clustering of Principal Components
HOG	Hight Osmolarity Glycerol
NaCl	Sodium Chloride
CYH	Cycloheximide
