# Supplementary material for: Characterization of Novel Lachancea thermotolerans Strains for Application in Table Olive Fermentation
Source: Foods. 2026 May 26;15(11):1883. doi: 10.3390/foods15111883 (PMC13257267; doi:10.3390/foods15111883)
Supplement: Supplementary file 1 [file foods-15-01883-s001.zip › SUPPLEMENTARY TABLE Foods.pdf]

SUPPLEMENTARY TABLE

Supplementary Table S1. Microorganisms used in this study and their ecological origin and geographic location.

Strain*	Species	Source	Geographic origin
BMA 8R1	*L. thermotolerans*	Viticultural soil	Southern Spain
BMA 45	*L. thermotolerans*	Mixed floral samples	Southern Spain
BMA 46	*L. thermotolerans*	Mixed floral samples	Southern Spain
BMA 48	*L. thermotolerans*	Arthropod-associated samples	Western Spain
BMA 50	*L. thermotolerans*	Fruit agroecosystem	Western Spain
BMA 54	*L. thermotolerans*	Mixed floral samples	Western Spain
BMA 56	*L. thermotolerans*	Fruit agroecosystem	Western Spain
BMA 57	*L. thermotolerans*	Fruit agroecosystem	Western Spain
BMA 60	*L. thermotolerans*	Mixed floral samples	Western Spain
BMA 61	*L. thermotolerans*	Fruit agroecosystem	Western Spain
BMA 63	*L. thermotolerans*	Fruit agroecosystem	Western Spain
BMA 64	*L. thermotolerans*	Mixed floral samples	Western Spain
BMA 65	*L. thermotolerans*	Mixed floral samples	Western Spain
BMA 66	*L. thermotolerans*	Fruit agroecosystem	Western Spain
BMA 70	*L. thermotolerans*	Fruit agroecosystem	Western Spain
BMA 71	*L. thermotolerans*	Mixed floral samples	Western Spain
BMA 72	*L. thermotolerans*	Mixed floral samples	Western Spain
BMA 122	*L. thermotolerans*	Fruit agroecosystem	Southern Spain
BMA 124	*L. thermotolerans*	Fruit agroecosystem	Southern Spain
BMA 125	*L. thermotolerans*	Viticultural soil	Southern Spain
BMA 127	*L. thermotolerans*	Fruit agroecosystem	Southern Spain
BMA 147	*L. thermotolerans*	Fruit agroecosystem	Southern Spain
BMA 166	*L. thermotolerans*	Arthropod-associated samples	Southern Spain
BMA 167	*L. thermotolerans*	Fruit agroecosystem	Southern Spain
BMA 180	*L. thermotolerans*	Viticultural soil	Western Spain
BMA 182	*L. thermotolerans*	Viticultural soil	Western Spain
BMA 183	*L. thermotolerans*	Viticultural soil	Western Spain
BMA 188	*L. thermotolerans*	Viticultural soil	Western Spain
BMA 189	*L. thermotolerans*	Fruit agroecosystem	Southern Spain
BMA 190	*L. thermotolerans*	Mixed floral samples	Southern Spain
BMA 191	*L. thermotolerans*	Arthropod-associated samples	Southern Spain
BMA 192	*L. thermotolerans*	Arthropod-associated samples	Southern Spain
BMA 193	*L. thermotolerans*	Arthropod-associated samples	Southern Spain
BMA 196	*L. thermotolerans*	Arthropod-associated samples	Southern Spain
BMA 214	*L. thermotolerans*	Mixed floral samples	Southern Spain
BMA 219	*L. thermotolerans*	Arthropod-associated samples	Southern Spain
BMA 221	*L. thermotolerans*	Fruit agroecosystem	Southern Spain

BMA 222	*L. thermotolerans*	Fruit agroecosystem	Southern Spain
BMA 223	*L. thermotolerans*	Arthropod-associated samples	Southern Spain
BMA 225	*L. thermotolerans*	Fruit agroecosystem	Southern Spain
BMA 11	*W. anomalus*	Spanish-style table olive fermentation	Southern Spain

Note*: Strain codes follow an internal collection system, where numbers indicate isolation order.
