# Supplementary figures and images for: Characterization of Novel Lachancea thermotolerans Strains for Application in Table Olive Fermentation
Source: Foods. 2026 May 26;15(11):1883. doi: 10.3390/foods15111883 (PMC13257267; doi:10.3390/foods15111883)

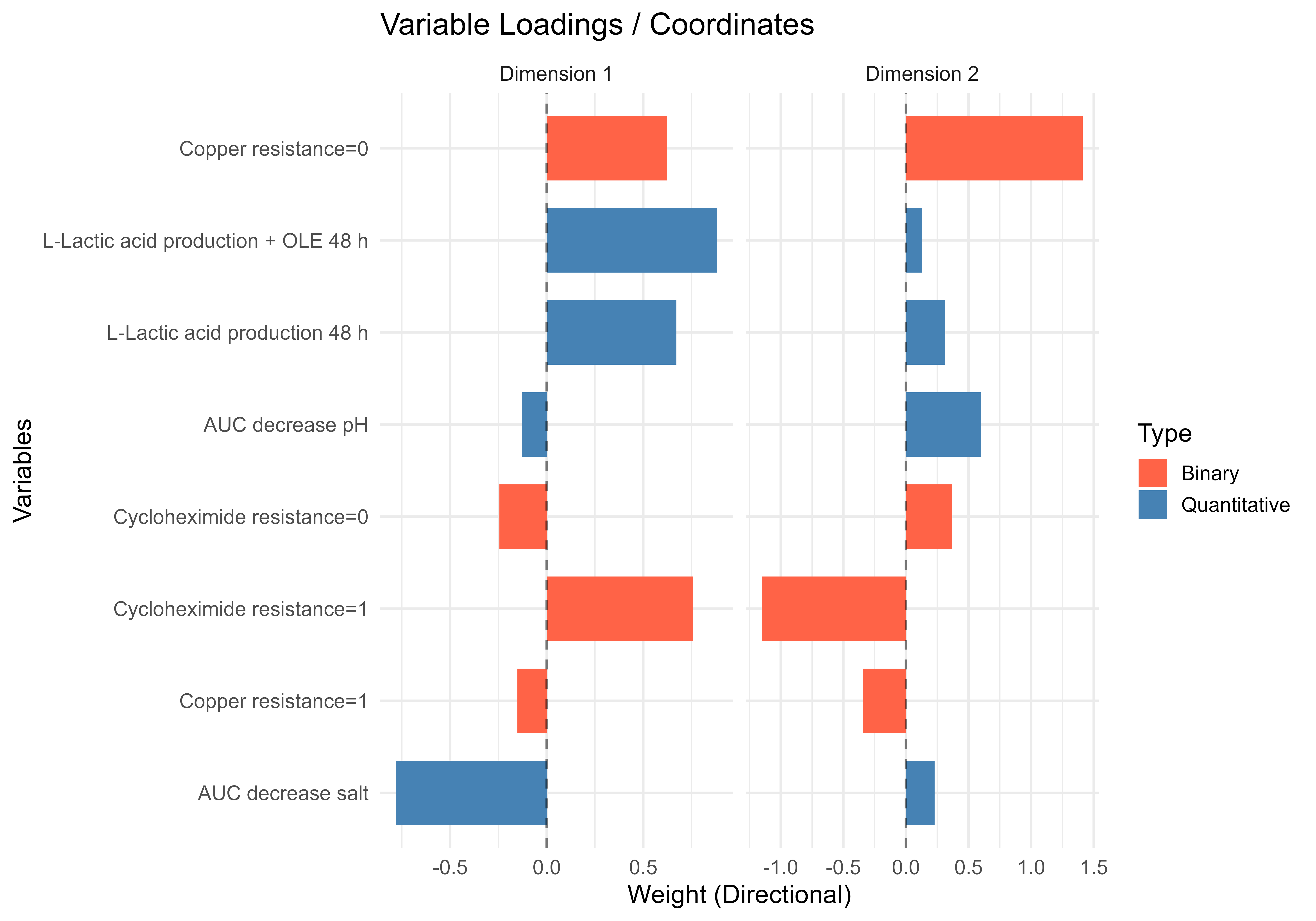

Supplement: Supplementary file 1 [file foods-15-01883-s001.zip › 3_Suppl Figure S1.tiff]
